# MRI signal and morphological alterations of the suprapatellar fat pad in asymptomatic subjects: are these normal variants?

**DOI:** 10.1007/s00256-022-04055-z

**Published:** 2022-04-15

**Authors:** Aurelio Cosentino, Raphaël Richard, Margaux Baron, Xavier Demondion, Julien Favre, Patrick Omoumi

**Affiliations:** 1 Hôpital Riviera-Chablais, Rennaz, Switzerland; 2 Department of Musculoskeletal Medicine, Swiss BioMotion Lab, Lausanne University Hospital and University of Lausanne, Lausanne, Switzerland; 3 Centre Hospitalier Régional Universitaire de Lille, Lille, France; 4 Department of Diagnostic and Interventional Radiology, Lausanne University Hospital and University of Lausanne, Lausanne, Switzerland

**Keywords:** Knee, MRI, Suprapatellar fat pad, Normal variants, Radiography, Impingement, Inflammation, Osteoarthritis

## Abstract

**Objective:**

To study the prevalence of suprapatellar fat pad (SPFP) MR alterations in asymptomatic subjects, in relation to a wide range of clinical/imaging parameters, including muscle performance tests and physical activity data.

**Materials and methods:**

We prospectively included 110 asymptomatic subjects as part of a cohort study. Inclusion criteria were no knee pain in the last year. Exclusion criteria were any medical/surgical history of a knee disorder. Subjects underwent knee and low-dose posture radiographs [EOS®], 3 T MRI, clinical examination including muscle performance tests, and physical activity monitoring. The presence/absence of SPFP alterations (hyperintensity and mass effect) were assessed through consensus reading on fluid-sensitive sequences. Differences between groups of knees with SPFP alterations and controls were tested for a total of 55 categorical/continuous clinical/imaging parameters, including SPFP relative-T2-signal, trochlear/patellar/lower-limb morphologic measurements. Wilcoxon-rank-sum and chi-square tests were used to compare groups of patients. The histological correlation was obtained in a cadaveric specimen.

**Results:**

SPFP alterations were common in asymptomatic subjects: hyperintensity 57% (63/110) and mass effect 37% (41/110), with 27% (30/110) showing both. Among the 55 imaging, clinical, or activity parameters tested, only increased patellar tilt angle (*p* = 0.02) and TT-TG distance (*p* = 0.03) were statistically different between groups of SPFP alterations and controls. The histological correlation showed more abundant connective tissue in SPFP compared to the prefemoral fat pad.

**Conclusions:**

SPFP hyperintensity and mass effect are common MRI findings in asymptomatic knees, and they are not related to most imaging, clinical, and activity parameters. Care should be taken not to overcall them pathological findings as they most likely represent normal variants.

## Introduction

The SPFP, or quadriceps fat pad, is one of the three intracapsular, extrasynovial fat pads of the knee. A normal SPFP is a triangular-shaped fat pad that fills the gap between the deepest layer of the quadriceps tendon insertion and the superior aspect of the patella. It is posteriorly lined by the synovium. It is thought to biomechanically improve the patellofemoral engagement of the extensor mechanism [1–5].

At knee MRI examinations, the SPFP can be increased in size or in signal intensity, the clinical significance of which alterations is not elucidated to date. Various studies on patient cohorts with different knee-related clinical conditions have theorized that these alterations may be either primary [2] or associated with anterior knee pain [2, 3, 6], overuse and repetitive microtrauma [2, 5], or development of knee osteoarthritis [7, 8].

The purpose of this study was to study the prevalence of SPFP MR alterations in asymptomatic subjects, in relation to a wide range of available clinical and imaging parameters, including anatomical knee and lower limb measurements, imaging signs of knee osteoarthritis, muscle performance, and physical activity. We hypothesized that these alterations are highly prevalent in asymptomatic subjects, with no correlation to joint or patient-related parameters.

## Materials and methods

### Population

We included subjects from an ongoing cohort study (Lausanne Knee Study) on asymptomatic knees, funded by the Swiss National Science Foundation (SNSF Grant #CRSII5_177155). This study was approved by the ethical committee of Canton de Vaud (project 2019-00291), and all participants provided written informed consent prior to enrolment in the study.

Dissections and histologic preparations were performed on a body that had been donated for scientific research to the Laboratory of Anatomy of the Centre Hospitalier Régional de Lille, France, in accordance with the ethical standards.

This study focuses on asymptomatic subjects from the general population from 18 to 70 years old, with the following inclusion criteria: no knee pain in the last year and no known osteoarthritis. Exclusion criteria are (i) suffering from an inflammatory joint disease, (ii) a life-threatening illness or (iii) neurological disorder/dementia, (iv) impaired gait pattern, (v) history of severe lower limb injury or surgery, (vi) having consulted a health professional for a lower limb issue in the last 3 months, (vii) wearing orthotics or using walking aids, (viii) body mass index ≥ 30, and (ix) general contraindications to non-contrast imaging studies. Additionally, the subcutaneous fat anterior to the SPFP region was assessed to ensure that all included exams had homogeneous fat signal saturation.

For each subject, one knee was randomly selected to perform radiographs and MRI according to the imaging protocol in Table 1.

**Table 1:** Imaging protocol

Radiographs • Standing right knee lateral, 30° flexion • Standing AP and LL low-dose posture radiographs (orbits to foot) [EOS System®]					
Knee MRI at 3 T • Proton-density weighted, turbo spin echo with fat saturation tri-planar (sagittal, coronal, axial) acquisition (PD TSE) • T1-weighted, 3D fast-turbo spin echo (T1 SPACE) • T2-weighted 3D dual-echo steady state (T2 DESS)					
MRI sequences parameters	PD TSE sagittal	PD TSE transverse	PD TSE coronal	T1 space	T2 DESS 3D
TR/TE	3980/38	4890/35	3980/35	700/11	12.32/4.43
Slice thickness (mm)	3	3	3	0.5	0.63
Interslice gap (mm)	0.3	0.3	0.3	-	-
Field of view (cm)	27.5 × 17	27.5 × 17	27.5 × 17	25.9 × 16	25.9 × 16
Matrix	768 × 768	448 × 408	448 × 392	320 × 304	256 × 240
Voxel (mm)	0.2. × 0.2 × 3.0	0.4 × 0.4 × 3.0	0.4 × 0.4 × 3.0	0.5 × 0.5 × 0.5	0.6 × 0.6 × 0.6
Excitation	1	1	1	1	1
Echo-train length	7	7	7	42	2
PAT factor	3	2	2	2	2

### Imaging and analysis

MR images were acquired using a dedicated 3.0 T scanner (Siemens 3 T MAGNETOM Prisma Fit, Siemens Healthcare, Erlangen, Germany) and a dedicated knee coil (Tx/Rx transmit/receive 15-channel, Siemens Healthcare), with non-weight-bearing knee in full extension.

### Imaging qualitative analysis

#### Suprapatellar fat pad and study groups

SPFP qualitative parameters (hyperintensity and mass effect) were evaluated independently by three board-certified radiologists A.C., R.R. et P.O. with 1, 6, and 11 years of experience in musculoskeletal radiology. Discordant cases were then reviewed to provide a consensus reading. The readers were blinded to morphological, demographic, and clinical information.

**Fig. 1:**
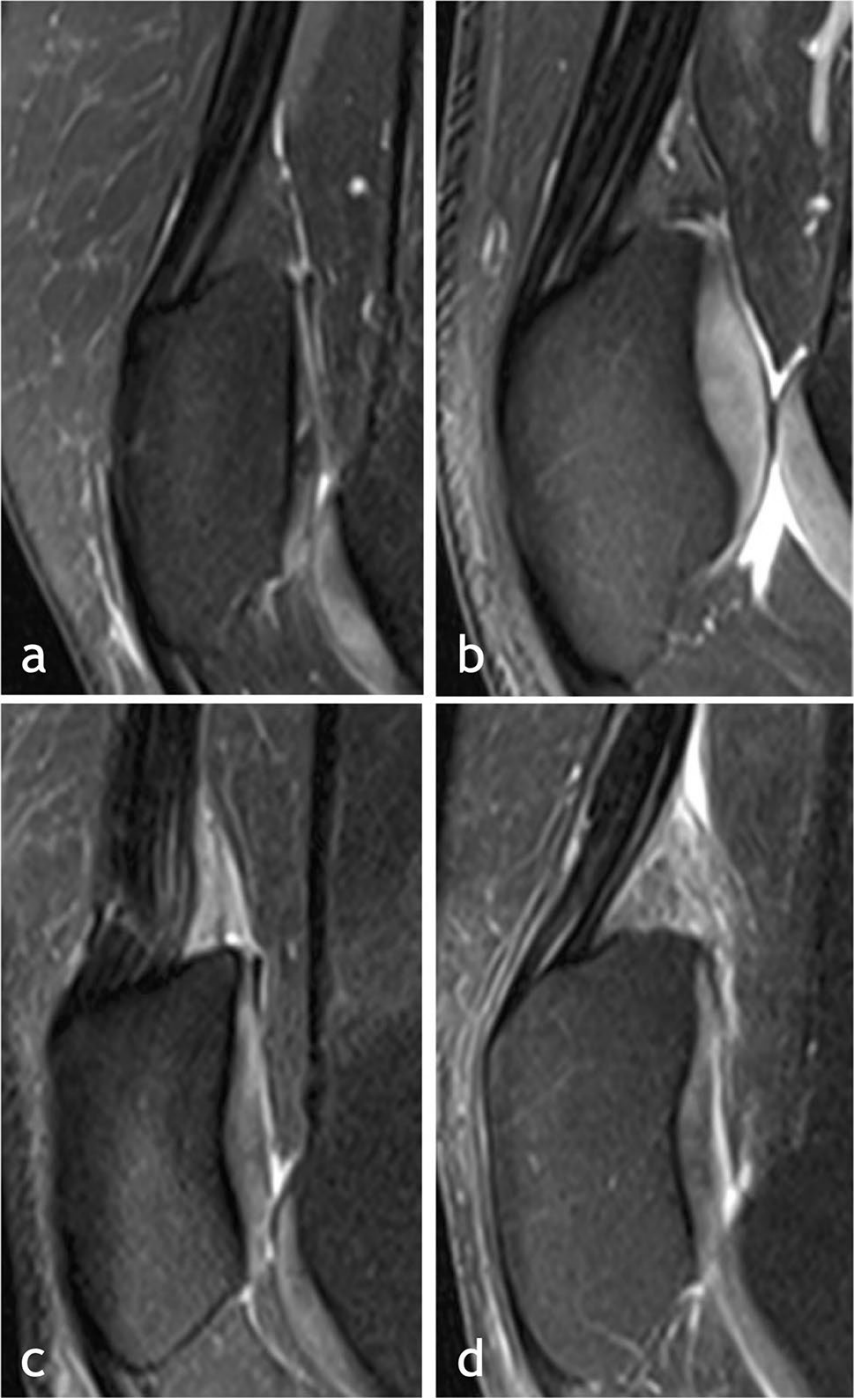
MRI 3 T midsagittal PD TSE fat-suppressed slices showing four different aspects of the suprapatellar fat pad. **a** No SPFP MRI alteration; **b** presence of SPFP mass effect only; **c** presence of SPFP hyperintensity only; and **d** presence of SPFP hyperintensity and mass effect

Qualitative alterations were assessed on one midsagittal PD TSE fat-suppressed slice (Fig. 1).

SPFP signal intensity was considered altered if higher than that of the prefemoral fat pad, as previously described [2, 3, 6, 7].

Mass effect was considered present if the posterior contour of the SPFP was convex, as previously described [2, 5]. The study population was divided into four groups based on the following characteristics of the SPFP qualitative assessment: SPFP signal alteration, SPFP mass effect, SPFP signal alteration and mass effect, and no signal or size alteration of the SPFP.

#### Osteoarthritis (OA)

Signs of OA on MRI were assessed in consensus by A.C. and P.O., according to the MRI definition of OA proposed by Hunter et al. [9], using 3D T1 SPACE, 3D T2 DESS and axial, coronal, and sagittal 2D PD TSE sequences (Table 1). Patellofemoral (PF) and tibiofemoral (TF) compartments were considered separately.

### Imaging quantitative analysis

Images were reviewed and analyzed on the institution’s PACS (Carestream VUE, Carestream Health, USA) by A.C. MRI signal intensity data and morphometric analysis reflecting knee joint and lower limb morphology were collected for each subject. A detailed description of these measures can be found in Table 2.

**Table 2:** Description of measurements

Axial evaluation—MRI	
Bicondylar line (BC line)	A line parallels the posterior margins of the femoral condyles
Trochlear axis (TA line)	A line connecting the anterior margins of the medial and lateral trochlear facets
Sulcus angle [10, 11]	The angle between the slopes of the lateral and medial facets Abnormal threshold for dysplasia: > 145°
Trochlear groove depth [10, 11]	The distance from the central deepest portion of the trochlear groove to the TA line Abnormal threshold for dysplasia: ≤ 4 mm
Trochlear medial and lateral facets length and asymmetry of the facet length [11, 12]	Asymmetry was calculated as the ratio of the medial-to-lateral facet length Abnormal threshold for dysplasia: ≤ 40%
Anteroposterior femoral distance (maximal medial/lateral condyles; minimal trochlear groove) [12]	The BC line is drawn. The distances drawn perpendicular to the BC line indicate the largest anteroposterior diameters of the lateral and medial trochlear facets and the deepest point of the sulcus
Lateral trochlear inclination [13]	The most superior slice showing trochlear cartilage is selected from the axial dataset. A line is drawn along the subchondral bone of the lateral trochlear facet, and the BC line is drawn. The lateral trochlear inclination is the angle between the two lines Abnormal threshold for dysplasia: < 11°
TT-TG [14, 15]	The BC line is drawn. Line 1 is perpendicular to the BC line and crosses the center of the trochlear groove in the slice of reference. Line 2 is perpendicular to the BC line and runs through the central part of the insertion of the patellar tendon on the distal image. The distance between lines 1 and 2 is TT-TG Abnormal threshold: > 20 mm
Patellar inclination (tilt) angle [16]	The BC line is drawn. Line 1 is drawn through the transverse axis of the patella. The patellar inclination is the angle between these two lines, positive values if it opens medially Abnormal threshold: > 10°
Lateral patellar displacement [12]	The shortest distance between the lateral margin of the trochlea and the lateral margin of the patella. Negative values were assigned if the patellar pole was medial to the lateral condyle Abnormal threshold: > 6 mm
Patellar width, medial and lateral facet length [11]; facet angle and asymmetry	Patellar facet asymmetry is the ratio of the medial and lateral patellar facet lengths. The so-called “odd facet” was not included in the medial facet measurement. Asymmetry was calculated as the ratio of the medial-to-lateral facet length
Sagittal evaluation—X-rays/MRI	
Patellar height [17]	Using the lateral radiograph of the knee, the following measurements are made: A. The most proximal articular margin of the patella to its most distal nonarticular aspect B. The most proximal to the most distal articular margin of the patella C. The length of the inner border of the patellar tendon from the lower pole of the patella to the notch in the tibial cortex at the superior border of the tibial tubercle (patellar tendon length) D. The most distal articular aspect of the patella to the notch in the tibial cortex at the superior border of the tibial tubercle E. The most distal articular aspect of the patella to the anterior margin of the tibial plateau
Insall–Salvati ratio	Distance ratio C/A
Modified Insall–Salvati ratio	Distance ratio D/B
Caton–Deschamps ratio	Distance ratio E/B
Patellar-trochlear overlap [3, 11]	Measures the length of the overlap between patellar and trochlear articulating cartilage on IRM
Patellar-trochlear index [3, 11]	Ratio: patellar articular height (B)/patellar-trochlear overlap Patella alta < 0.18
Ventral trochlear prominence [12]	The distance between the line paralleling the ventral cortical surface of the distal femur ant the most ventral cartilaginous point of the femoral trochlear floor Abnormal threshold: > 8 mm
Crossing sign [14]	The crossing sign is positive when the contours of the trochlear floor and of the lateral femoral condyle intersect at any level
Trochlear beak/spur [12]	The trochlear beak, or spur, refers to an angular projection of the most proximal portion of the trochlea
EOS	
Femoral-tibial angles	Varus/valgus angles were measured on AP projections Flessum/recurvatum angles were measured on LL projections
Q angle [18]	The line joining the anterior superior iliac spine (ASIS) and the midpoint of the patella was drawn. The line joining the midpoint of the patella and the tibial tuberosity was drawn. The angle between these lines is the Q angle Abnormal threshold: > 20°
Dysmetria	The difference between the functional length of lower limbs [= study’s lower limb—contralateral]

#### MRI – suprapatellar and Hoffa’s fat pad

Anteroposterior, craniocaudal, and oblique diameters [5] and total volume of SPFP were measured. Anteroposterior and craniocaudal diameters and total volume of Hoffa’s fat pad were measured. The presence of superolateral Hoffa’s fat pad edema was recorded.

**Fig. 2:**
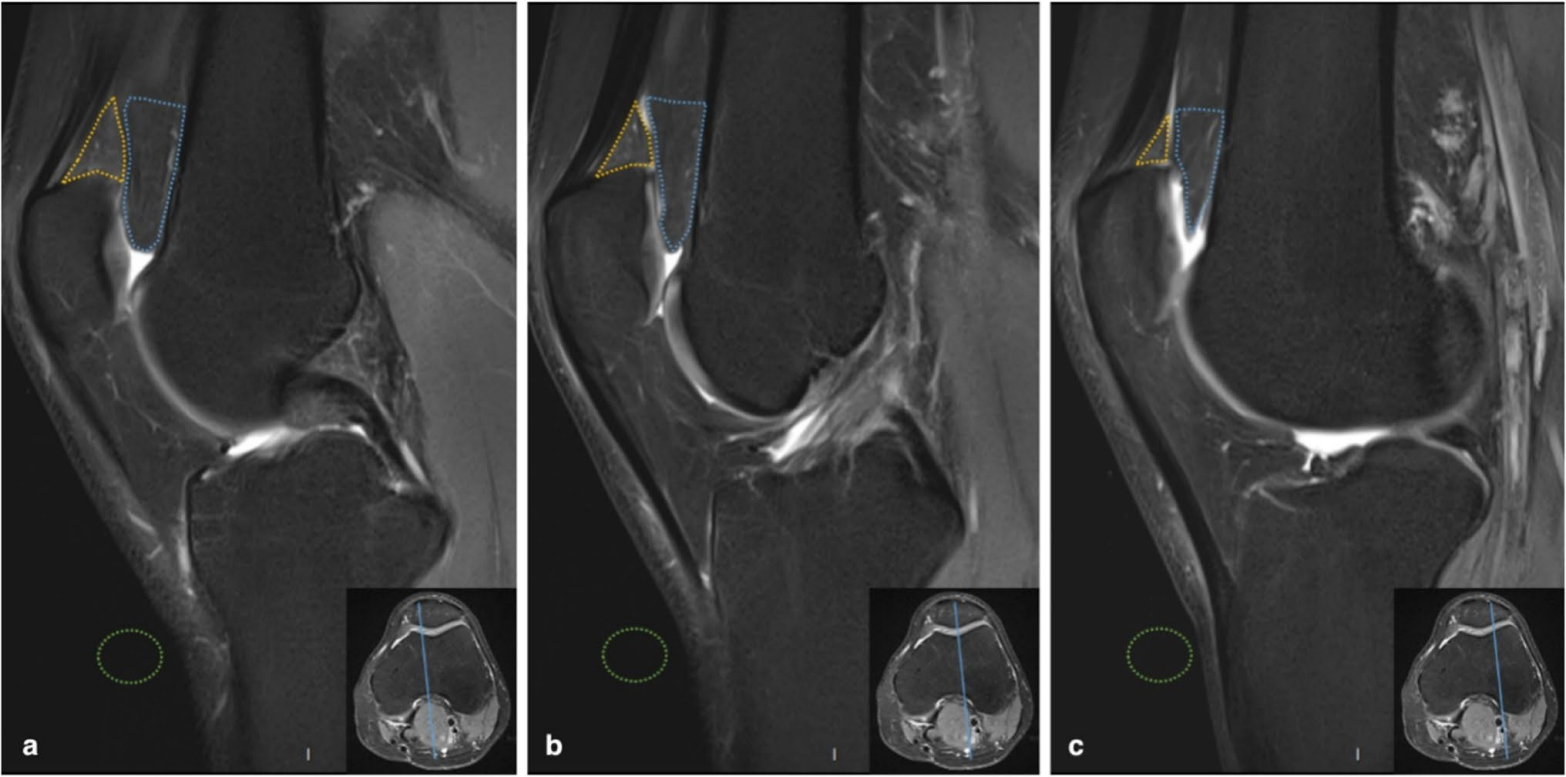
MRI 3 T sagittal PD TSE fat-suppressed slices. SPFP relative signal intensity was measured in three slices at the midpoint of the medial trochlear facet (**a**), at the center of the trochlea (**b**), and at the midpoint of the lateral trochlear facet (**c**). For each slice, three regions of interest (ROIs) were drawn: one around the SPFP (yellow), one around the prefemoral fat pad (blue) at the same level, and one in the air (green) in the lower part of the image for the background noise

SPFP signal intensity was measured on three different sagittal images, at the center of the trochlea and at the midpoint of the lateral and medial trochlear facets (Fig. 2). For each slice, three regions of interest (ROIs) were drawn: one around the SPFP, one around the prefemoral fat pad at the same level, and one in the air in the lower part of the image for the background noise. The SPFP relative signal intensity was calculated for each slice as SPFP relative signal intensity = signal intensity difference (SPFP value − PFP value)/background noise SD. The mean of the three measurements was used as the final relative SPFP signal intensity [5].

#### MRI/X-rays/EOS—trochlear morphology and patello-femoro-tibial-alignment

**Fig. 3:**
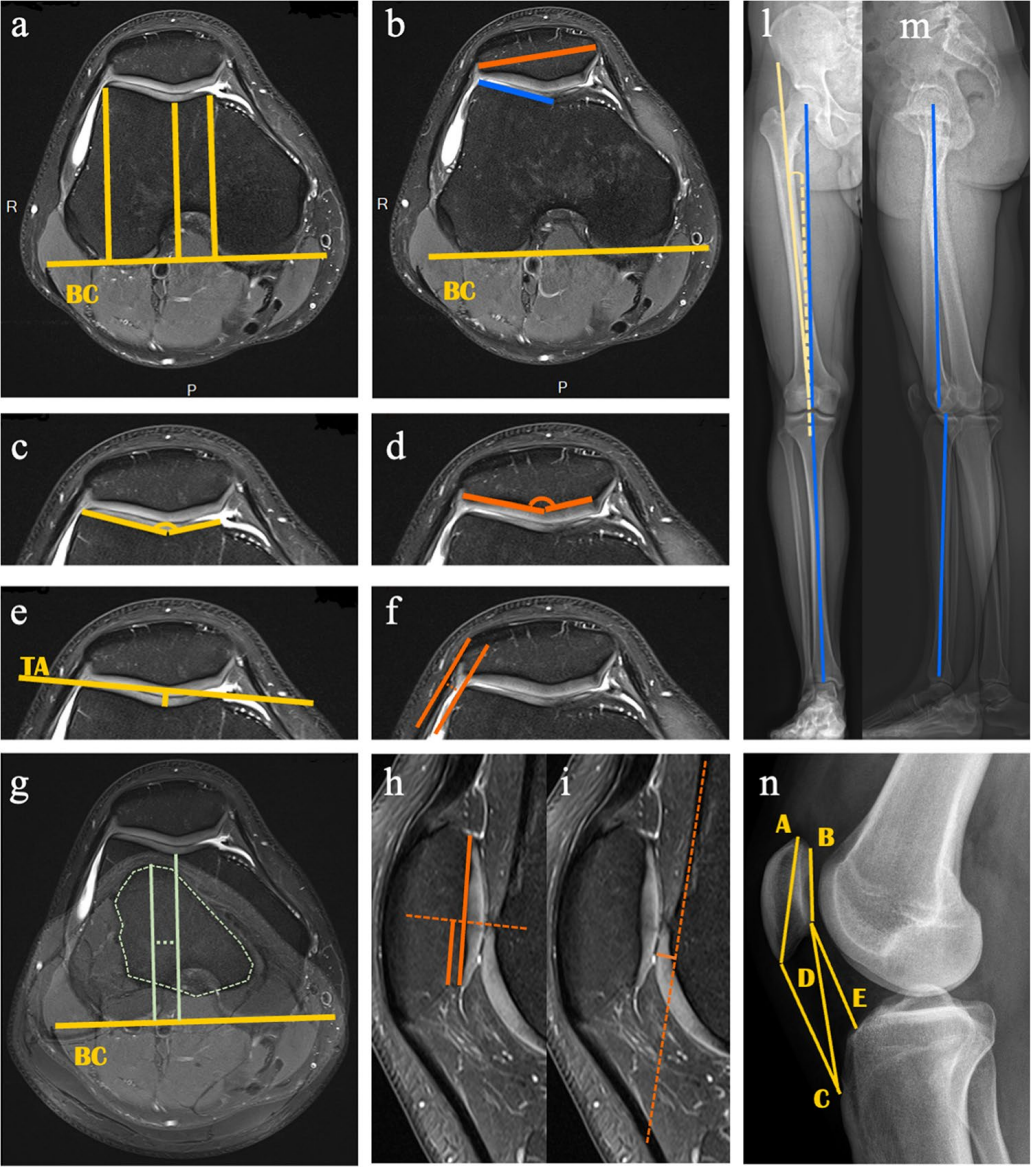
Images of performed measurements on 3 T MRI, EOS, lateral X-rays. A detailed description can be found in Table 2. Bicondylar line (BC line) (**a**, **b**, **g**); anteroposterior femoral distance (maximal medial/lateral condyles; minimal trochlear groove) (**a**); lateral trochlear inclination (blue) and patellar width and inclination angle (orange) (**b**); trochlear medial and lateral facets length and asymmetry of the facet length and sulcus angle (**c**); patellar medial and lateral facet length, facet angle and asymmetry (**d**); trochlear axis (TA line) and trochlear groove depth (**e**); lateral patellar displacement (**f**); TT-TG (**g**); patellar height, patellar-trochlear overlap and index (**h**); ventral trochlear prominence (**i**); femoral-tibial angles (**l**, **m**); Q angle (**l**); Insall–Salvati ratio, modified Insall–Salvati ratio, Caton–Deschamps ratio (**n**)

Several indices for trochlear morphology and femoropatellar joint analysis were measured (Table 2 and Fig. 3). Trochlear morphologic characteristics were assessed on axial PD images established at the slice in which trochlear articular cartilage spanned the entire trochlear surface [10, 13, 19]. Patellar morphologic characteristics were assessed on axial images at the slice of maximum patellar width. On a standard knee lateral projection, the Insall–Salvati index, the modified Insall–Salvati index, and the Caton–Deschamp index were calculated, and the presence of a cross sign was recorded. On EOS, the lower limb functional length, two-plane femorotibial alignment angles (varus/valgus and flessum/recurvatum), and the Q angle were measured; dysmetria was calculated.

When possible, the geometrical variables were analyzed not only as continuous but also as dichotomic variables, applying threshold values commonly accepted and published in the literature.

### Subjects’ clinical evaluation and muscle performance

The patient’s characteristics (age, gender, ethnicity, height, weight, BMI, abdominal circumference) and habits (smoking, alcohol consumption) were recorded on the day of testing. A clinical evaluation of the knee was performed by a certified physiotherapist (M.B.) analyzing knee stability, range of motion, and presence of crepitus. In addition, participants performed two sets of three measures of isokinetic concentric knee flexion and extension at 60°/s and 180°/s, respectively, using a dynamometer (CYBEX, Computer Sports Medicine, MA). Participants performed a warm-up trial at 50% maximal effort for each testing speed. A rest period of 30 s was provided between the warm-up and testing sets, and a rest period of 90 s was given between the 60 and the 180°/s tests. Concentric, isokinetic peak torque was defined as the single highest torque output recorded throughout the range of movement of each set. The product of the moment and actual angular velocity derived from the angular displacement data was also used to measure the peak power for each set, thus evaluating performances for hamstrings (in knee flexion) and quadriceps (in knee extension) muscles.

### Subjects’ physical activity

Participants’ physical activity was measured with two methods. First, the International Physical Activity Questionnaire (IPAQ) was used to categorize participants in low/moderate/high activity groups. Second, participants were given a wrist-worn device (GENEActiv Original, Activinsights, UK), which provided sedentary time (minutes/week) and time spent in moderate and vigorous physical activity (minutes/week) for 14 consecutive days.

### Histologic correlation

The anatomical and histological correlation was obtained in one cadaveric specimen (65-year-old male), not part of the imaging study.

### Statistical analysis

Data were analyzed using Matlab (release R2019b, The MathWorks Inc., Natick, MA) using Wilcoxon rank-sum test for continuous variables and the chi-square test for dichotomic variables on four distinct groups (SPFP alterations vs. controls). An a priori alpha level of 5% was used, and a Bonferroni correction was applied to adjust for multiple testing.

## Results

### Cohort characteristics

One hundred and ten (110) asymptomatic subjects were included in the study (mean age 34.1 years, range 18–68 years); 51 were male subjects. Subjects had a mean height of 171.7 cm (range 150–193 cm), a mean weight of 70.9 kg (range 49–107 kg), a mean BMI of 23.9 (range 18–31.6), and a mean abdominal circumference of 78.9 cm (range 62–105 cm). Ethnicity distribution was as follows: 4 African subjects (4%), 2 Asian subjects (2%), 84 Caucasian subjects (76%), 6 Hispanic subjects (5%), and 14 mixed/other subjects (13%). Alcohol consumption (1 unit/week or more) was declared by 76 subjects (69%), and 12 patients (11%) had smoking habits.

Osteoarthritis at MRI was identified in 20% (22/110) of asymptomatic subjects in the PF compartment and in 5.5% (6/110) in the TF compartment, 2/110 showed both PF and TF OA. Up to 20% (22/110) of subjects showed only some sign of osteoarthritis without fulfilling all MRI OA criteria; 58% (64/110) of subjects showed no signs of osteoarthritis.

### Qualitative analysis

#### SPFP alterations

Suprapatellar fat pad MRI alterations were common in asymptomatic subjects: 57% (63/110) of subjects showed hyperintensity and 37% (41/110) mass effect, with 27% (30/110) of subjects showing both.

#### Osteoarthritis

No statistically significant difference in the presence of MRI signs of OA between groups of SPFP alterations and controls was found.

### Quantitative analysis

The number of subjects in the four mutually exclusive groups for the quantitative analysis was SPFP hyperintensity (*n* = 33), SPFP mass effect (*n* = 11), SPFP hyperintensity and mass effect (*n* = 30), and controls = 36 with no alterations.

The following measurements are expressed as median (first quartile Q1; third quartile Q3).

#### MRI—quantitative vs. qualitative SPFP alterations (Table 3)

Relative hyperintensity measurements were found to be significantly different between the group with hyperintensity and mass effect and the control group (*p* = 0.04 (30.8 (19.2; 56.7) vs. 14.7 (7.3; 31.6)), respectively). Mass effect measurements were found also to be significantly different between the group with hyperintensity and mass effect and the control group: SPFP anteroposterior diameter, *p* = 0.04 (7.9 mm (7;8.6) vs. 6.8 mm (5.2;8.3)); SPFP oblique diameter, *p* = 0.002 (11 mm (9.6;12.1) vs. 8.7 mm (7.1;10.7)); SPFP volume, *p* = 0.01 (1.9 cm^3^ (1.5;2.6) vs. 1.4 cm^3^ (1.1;1.8)). No statistically significant difference was found with the SPFP cranio-caudal diameter.

**Table 3:** Quantitative vs. qualitative SPFP alterations

	Control (*n* = 36)	Mass effect (*n* = 11)	Hyperintensity (*n* = 33)	Hyp&Mass (*n* = 30)	Con vs. Mass	Con vs. Hyp	Con vs. Hyp&Mass
	Median (Q1;Q3)	Median (Q1;Q3)	Median (Q1;Q3)	Median (Q1;Q3)	*p*-value	*p*-value	*p*-value
SPFP_Diameter_AP [mm]	6.8 (5.2;8.3)	8.9 (7.4;9.2)	7.4 (6.5;9.1)	7.9 (7;8.6)	0.10	0.75	**0.04**
SPFP_Diameter_OBL [mm]	8.7 (7.1;10.7)	10.9 (9.5;12)	9.6 (8.5;11.5)	11 (9.6;12.1)	0.08	0.24	**0.002**
SPFP_Diameter_CC [mm]	12.7 (11;14.2)	12.2 (10.7;12.7)	13.1 (11.8;14.7)	12.9 (11.4;15)	1.00	0.60	1.00
SPFP_Volume [cm^3^]	1.4 (1.1;1.8)	1.6 (1.5;1.9)	1.6 (1.3;2.1)	1.9 (1.5;2.6)	0.76	0.36	**0.01**
SPFP_Relative_Signal	14.7 (7.3;31.6)	19.2 (4.7;26.1)	31.7 (13.5;45.6)	30.8 (19.2;56.7)	1.00	0.21	**0.04**

Imaging SPFP MRI variables: statistical analysis on continuous variables between four groups based on different SPFP MRI appearances. (*Con*, controls; *Mass*, mass effect only; *Hyp*, hyperintensity only; *Hyp&Mass*, hyperintensity and mass effect; *OA*, osteoarthritis; *AP*, anteroposterior; *CC*, cranio-caudal; *OBL*, oblique diameters. Bold values represent statistically significant differences (*p* < 0.05)

#### MRI/X-rays/EOS—trochlear morphology and patello-femoro-tibial-alignment (Table 4)

Among the variables tested, only two measurements proved statistically significantly different between the groups: patellar tilt angle was significantly higher in the group with mass effect compared to controls (*p* = 0.02 (14.4° (11.5;17.9) vs. 9.7° (5.8;13.1))), and TT-TG distance was significantly higher in the group with hyperintensity and mass effect than in the control group (*p* = 0.03 (11.6 mm (9.1;14.3) vs. 9.3 mm (7.1;11.8))). There was no statistically significant difference between groups in the dichotomic analysis by applying previously published abnormal thresholds, including for the patellar tilt (*p* = 0.06) and TT-TG (no patient with abnormal values > 20 mm).

**Table 4:** Imaging X-rays/EOS/MRI variables

		Continuous variables							Dichotomic variables		
		Control (*n* = 36)	Mass effect (*n* = 11)	Hyperintensity (*n* = 33)	Hyp&Mass (*n* = 30)	Con vs. Mass	Con vs. Hyp	Con vs. Hyp&Mass	Con vs. Mass	Con vs. Hyp	Con vs. Hyp&Mass
		Median (Q1;Q3)	Median (Q1;Q3)	Median (Q1;Q3)	Median (Q1;Q3)	*p*-value	*p*-value	*p*-value	*p-*value	*p*-value	*p*-value
Age (years)		28.5 (24;40)	27 (23;30)	32 (24.5;54.5)	27 (25;31)	1.00	0.65	1.00			
Hoffa’s fat pad	Hoffa’s fat pad AP diameter [mm]	31.4 (28.9;33.9)	30.8 (26.7;37.4)	30.7 (27.3;34)	33.5 (29;35.1)	1.00	1.00	0.74			
	Hoffa’s fat pad CC diameter [mm]	47.2 (44.2;51.9)	49.8 (45.1;53.7)	47.3 (43.7;50.9)	49.4 (44.8;53)	1.00	1.00	1.00			
	Hoffa’s fat pad volume [cm^3^]	25.7 (20.3;30.3)	28.5 (22.5;31.8)	23.7 (20.9;29.4)	28.4 (21.6;32.3)	1.00	1.00	0.53			
OA	Tibifemoral OA								0.42	0.57	0.67
	Patellofemoral OA								0.09	0.84	0.83
MRI axial morphometric evaluation	Trochlear lateral facet length [mm]	22.6 (20.2;24.4)	22.1 (20.8;24.1)	23 (21.4;24.8)	24.2 (22;25.8)	1.00	1.00	0.13			
	Trochlear medial facet length [mm]	12.8 (11.6;14.6)	11.7 (10.9;13.6)	12.2 (11.2;15)	13.1 (11.8;14.3)	1.00	1.00	1.00			
	Trochlear asymmetry	0.6 (0.5;0.6)	0.5 (0.5;0.6)	0.5 (0.5;0.6)	0.5 (0.5;0.7)	1.00	1.00	0.54	0.67	0.61	0.67
	Trochlear sulcus angle [°]	139.3 (134.6;146.1)	144.2 (136.8;150.3)	141.3 (134.6;147.5)	142.1 (136.8;144.4)	0.64	1.00	1.00	0.36	0.80	0.19
	Trochlear depth [mm]	5.3 (4.3;6.2)	4.7 (3.9;5.8)	4.9 (4.2;6.2)	5.2 (4.5;6)	0.94	1.00	1.00	0.19	0.25	0.51
	Condilar lateral length [mm]	62.7 (60.2;67.2)	63.9 (62.4;68)	61.2 (59.2;67.4)	64.4 (61.1;66.2)	0.93	1.00	1.00			
	Minimal trochlear groove length [mm]	55.6 (53;59.2)	57.5 (56.2;58.6)	54.5 (51.7;58.2)	57.6 (54.3;59.7)	1.00	1.00	0.81			
	Condilar medial length [mm]	59.2 (56.6;63.6)	62.2 (60.2;62.5)	57.1 (56.2;62.5)	62.6 (57.8;65.7)	0.65	1.00	0.36			
	Lateral trochlear inclination [°]	18.4 (15.8;22.3)	14.9 (11.7;19)	20.1 (15.7;24.1)	17.1 (14.3;22.1)	0.21	1.00	1.00	0.35	0.60	0.80
	TT-TG [mm]	9.3 (7.1;11.8)	9.9 (8.5;10.1)	7.9 (5.1;10.5)	11.6 (9.1;14.3)	1.00	0.17	**0.03**			
	Patellar lateral facet length [mm]	24.9 (23.1;26.7)	24.2 (22.6;26.1)	25.1 (23.2;28.6)	27.4 (25.4;29)	1.00	1.00	0.05			
	Patellar medial facet length [mm]	14 (12.5;15.3)	13.6 (12.4;15.2)	14 (12.5;15.7)	13.7 (12.7;15.5)	1.00	1.00	1.00			
	Patellar width [mm]	42.6 (40.2;45.6)	42.3 (41;42.9)	41.2 (38;45.4)	43.5 (41.2;45.8)	1.00	1.00	1.00			
	Patellar asymmetry	0.6 (0.5;0.6)	0.6 (0.5;0.7)	0.5 (0.5;0.6)	0.5 (0.4;0.6)	1.00	1.00	0.15			
	Patellar_angle [°]	128.3 (124.1;132.6)	129.1 (123.4;132.3)	127 (122.7;131.4)	126.6 (122;129.3)	1.00	1.00	0.76			
	Patellar tilt [°]	9.7 (5.8;13.1)	14.4 (11.5;17.9)	10.9 (7.5;12.7)	12.4 (6.2;15.3)	**0.02**	1.00	0.80	0.06	0.71	0.59
	Patellar lateral displacement [mm]	− 2.9 (− 4.3;0.5)	0 (− 2;1.8)	− 2.1 (− 3.5;1.6)	− 0.9 (− 3.3;1.7)	0.31	1.00	0.45			
	Patellar volume [cm^3^]	18.9 (16.5;22.6)	19.4 (18.2;23)	18.9 (16.6;22.8)	21.4 (18.2;25.3)	1.00	1.00	0.52			
RX/MRI sagittal and EOS morphometric evaluation	Insall–Salvati ratio	1.1 (1;1.3)	1.2 (1.1;1.3)	1.1 (1;1.3)	1.1 (1;1.2)	0.42	1.00	1.00	0.28	0.81	0.89
	Modified Insall–Salvati ratio	1.7 (1.7;1.9)	1.8 (1.7;1.9)	1.8 (1.6;1.9)	1.8 (1.6;1.9)	1.00	1.00	1.00	0.68	0.28	0.32
	Caton–Deschamps ratio	1.1 (1;1.1)	1.1 (1;1.1)	1 (0.9;1.1)	1 (0.9;1.2)	1.00	0.06	0.69	0.67	0.17	0.25
	Patellotrochlear overlap [mm]	9.2 (7.1;11.9)	8.7 (7.2;10)	9.3 (6.4;10.9)	9.5 (7.7;11.6)	1.00	1.00	1.00			
	Patellotrochlear index [mm]	0.3 (0.2;0.3)	0.3 (0.2;0.3)	0.3 (0.2;0.3)	0.3 (0.2;0.3)	1.00	1.00	1.00	0.25	0.25	0.32
	Ventral trochlear prominence [mm]	4.4 (3.6;5.9)	4.2 (3.4;4.9)	4.4 (2.9;5.3)	4.8 (3.8;5.5)	0.77	0.70	1.00	0.58	0.33	0.36
	X-ray-crossing sign								0.83	0.13	0.10
	X-rays-throclear beak/spur								0.44	0.79	0.54
	EOS-recurvatum/flessum angle [°]	4 (2;5.8)	5 (1.3;8.3)	3 (1;5.3)	2 (0;4)	1.00	1.00	0.11			
	EOS-varus/valgus angle [°]	− 1 (− 2;0.8)	0 (− 0.8;1)	− 1 (− 2;2.3)	0 (− 1;2)	1.00	1.00	0.46			
	EOS-Q angle′ [°]	175.6 (173.4;177.3)	175.8 (174.2;176.8)	175.1 (173.8;177)	175.7 (173.9;177.1)	1.00	1.00	1.00			
	EOS dismetry [mm]	0 (− 0.3;0.4)	− 0.3 (− 0.4;0.3)	0 (− 0.3;0.3)	0.2 (− 0.2;0.5)	1.00	1.00	0.45			

Imaging X-rays/EOS/MRI variables: statistical analysis on continuous and dichotomic variables between four groups based on different SPFP MRI appearances. (*Con*, controls; *Mass*, mass effect only; *Hyp*, hyperintensity only; *Hyp&Mass*, hyperintensity and mass effect; *OA*, osteoarthritis; AP, anteroposterior; *CC*, cranio-caudal. Bold values represent statistically significant differences (*p* < 0.05)

None of the geometrical variables showed any statistically significant differences between groups at dichotomic analysis.

No statistically significant difference was found between the groups regarding Hoffa’s fat pad measurements.

#### Subjects’ clinical evaluation, muscle performance, and physical activity quantification

No clinical evaluation, muscle performance test, or physical activity measurement, monitored for 2 weeks, was different between groups of SPFP alterations.

### Histopathological correlation

**Fig. 4:**
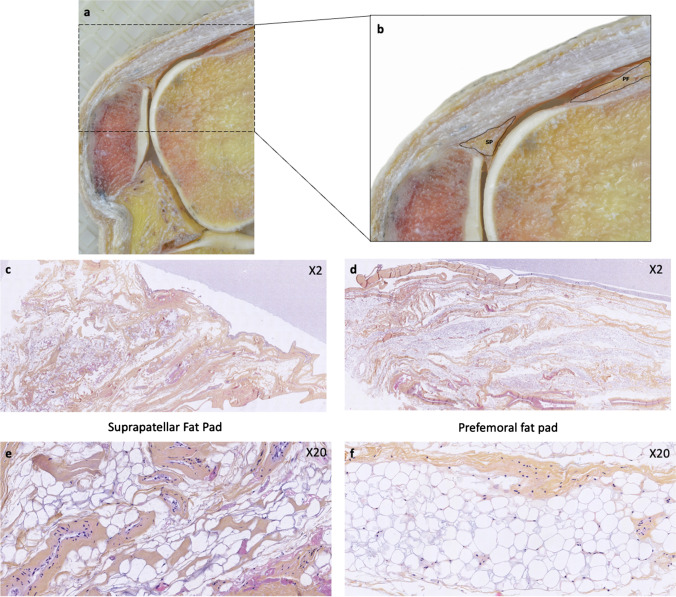
Cadaveric specimen (65-year-old male), not included in the imaging study. **a**, **b** Sagittal macroscopic section of a knee in a cadaveric specimen, suprapatellar fat pad, and prefemoral fat pad are contoured. **c**–**f** Hematoxylin and eosin-stained histological slices. **c**, **d** The suprapatellar fat pad shows the presence of more prominent strands of fibroconnective tissue traversing the fat, in comparison to the simple lobulated prefemoral fat pad (**d**, **f**)

The histological correlation on the cadaveric specimen highlighted differences in the structure of the SPFP compared to the prefemoral fat pad (PFP). The SPFP showed the presence of more prominent strands of fibroconnective tissue traversing the fat, in comparison to the simple lobulated prefemoral fat pad (Fig. 4).

## Discussion

In previous studies on the suprapatellar fat-pad alterations, investigators have focused on correlating the presence of SPFP mass effect and hyperintensity on proton-density fat-suppressed MRI sequences mainly with signs of patellofemoral malalignment, osteoarthritis, and knee pain, with equivocal conclusions in regard to their pathogenesis and clinical significance [2, 3, 5, 6, 8].

Based on the assumption that the pathophysiology of SPFP lesions is similar to that of the infrapatellar fat alterations, the dominant idea in the literature is that an abnormal SPFP may represent ongoing inflammatory processes, which may eventually damage the joint structures [2, 5].

In this study, we assessed the prevalence of SPFP signal and mass effect at MRI in healthy asymptomatic volunteers and compared a number of clinical and imaging parameters between groups of knees with SPFP MRI alterations and controls. This study was carried out in a research setting, giving us access to a controlled and homogeneous group of subjects selected for their absence of symptoms, as well as systematic data collection.

We showed that SPFP hyperintensity on fat-suppressed proton density-weighted sequences is very common and was present in more than half (56.4%) of our asymptomatic subjects, a finding that is in agreement with those of Roth et al. [2], Wang et al. [6], and Schwaiger et al. [7] (range 42–54%).

The convex appearance of the posterior border of the SPFP (mass effect) was present in 37% of our study population, a percentage much higher than previously reported (range 10–13%) [2, 3, 5–7]. The increased patellar tilt angle (median, 14.4°) was found to significantly differ between the group with SPFP mass effect and controls. This measurement lies in the pathological range (patellar tilt values are considered normal when < 10° [14] and averaged 9.7° in our control group (no SPFP abnormality present)). The TT-TG distance was also significantly different in the group with SPFP hyperintensity and mass effect compared to controls. The TT-TG was 2 mm greater in the group with SPFP hyperintensity and mass effect (median, 11.6 mm) than in controls (median, 9.3 mm), but these values lay in the normal range (TT-TG measurements are considered normal < 20 mm). A cause-and-effect relationship between patellar tilt and TT-TG distance and the presence of SPFP MRI alterations seems unlikely. First, patellar tilt and TT-TG were statistically different between groups in the continuous analysis but not in the dichotomic analysis. Second, previous reports did not find any correlation between these parameters and SPFP alterations. Indeed, Schwaiger et al. [7] did not find any correlation between increased patellar tilt angle and SPFP hyperintensity, while Tsavalas et al. [5] did not find any correlation between increased TT-TG distance and SPFP mass effect.

In agreement with previous reports [2, 5, 6], the vast majority of knees with SPFP mass effect showed also increased MRI signals (73%).

The cause of SPFP MRI alterations remains debated. Excessive knee flexion angles and overuse have been suggested as a cause of SPFP “inflammation” and enlargement leading to knee pain [2, 6]. We studied a possible association between SPFP alterations and functional knee measurements, regarding range of motion, muscle performance, and physical activity evaluation over 2 weeks and found no statistically significant difference between groups.

Another potential theory for SPFP alterations is that these changes are associated with the development of OA [6–8]. We did not find any statistically significant difference in the prevalence of MRI signs of OA between groups of knees with and without SPFP MRI alterations.

The important role of fat pads as packing tissue in synovial joints has been previously described [1–5]. Indeed, configurational changes occur in the joint during movement, in particular, the angle at which tendons and ligaments attach to bones and fat pad volumes adapt to these changes. The histological structure of the reservoir of fat on the surface of tendons has been characterized and referred to as insertional angle fat [20], forming part of the enthesis organ [21], such as that associated with the Achilles tendon [20, 22], the distal insertion of the patellar tendon [20], or at the entheses of the fibularis longus and brevis tendons [20]. The insertional angle fat at these anatomical sites generally presents increased blood supply and a greater amount of lamellated corpuscles and fibrous tissue compared to simple fat, and it is thought to play a role in the mechanosensory function at entheses [20, 23]. At histological analysis, we also found that the structure of the SPFP was different from that of the prefemoral fat pad. Specifically, more abundant connective tissue was visible in the SPFP than in the prefemoral fat pad, not only at histology but also in gross dissections (Fig. 4). The SPFP could therefore be considered a large insertional angle fat pad [4, 20] at the junction of the quadriceps tendon and patellar bone, and the variable MRI signal that we have observed in asymptomatic subjects could be related to its normal histological structure.

Our study presented some limitations. First, only the knees of asymptomatic patients were included in the study, and any direct comparison could be made with knees suffering from specific conditions (for example known patellofemoral malalignment, osteoarthrosis, specific athletes’ groups, and overuse clinical conditions). Second, our study focused on the prevalence of SPFP alterations at a single time point, and it is not clear how these alterations may evolve over time. Third, no biopsy was available on the imaged population for histopathological correlation, and only one cadaveric specimen was examined; more specific studies on the SPFP histopathological-MRI appearance correlation are therefore required. Fourth, we used a consensus reading instead of independent readings, which has several disadvantages including the fact that it is not representative of clinical practice, that it often represents the most outspoken or most experienced readers, and that it does not allow the assessment of interobserver variability. However, it was not our goal to reflect the interobserver variability in reporting these alterations in clinical practice. Our goal was to verify the presence of these alterations and the general validity of our findings through confirmation by more than one observer, in which case consensus reading may be an accepted method [24].

## Conclusion

In conclusion, we have described the normal MRI aspect of the SPFP in a controlled asymptomatic population and showed that SPFP high signal abnormality and mass effect are common findings in knee MRI of asymptomatic subjects (57% and 37%, respectively). Except for two parameters related to patellofemoral morphology, their presence was not related to any of the numerous morphological and functional variables tested. Therefore, these findings likely represent normal variants, and care should be taken not to overcall them pathological findings in clinical practice.
